# Entomopathogenic Activity of a Variety of the Fungus, *Colletotrichum acutatum*, Recovered from the Elongate Hemlock Scale, *Fiorinia externa*

**DOI:** 10.1673/031.009.1301

**Published:** 2009-04-16

**Authors:** José A. P. Marcelino, Svetlana Gouli, Bruce L Parker, Margaret Skinner, Rosanna Giordano

**Affiliations:** ^1^ Department of Plant and Soil Science, Entomology Research Laboratory, The University of Vermont, Burlington, VT 05405-0105 USA; ^2^ Illinois Natural History Survey, Division of Biodiversity and Ecological Entomology, Champaign, IL 61820 USA

**Keywords:** fungal epizootic, *Tsuga canadensis*, *Orthezia praelonga*

## Abstract

A fungal epizootic in populations of *Fiorinia externa* Ferris (Hemiptera: Diaspididae) infesting hemlock trees, *Tsuga canadensis* (L.) Carrière (Pinales: Pinaceae) in forests of the Northeastern US has been recently detected. The current known distribution of the epizootic spans 36 sites in New York, Pennsylvania, New Jersey and Connecticut. *Colletotrichum acutatum* Simmonds var. *fioriniae* Marcelino and Gouli var. nov. inedit. (Phyllachorales: Phyllachoraceae) was the most prevalent fungus recovered from infected scales. Bioassays indicated that this *C*. *acutatum* variety is highly pathogenic to *F*. *externa*. Mortality rates of >90 and >55% were obtained for *F*. *externa* crawlers and settlers, respectively. Significantly lower mortality levels, ≤ 22%, were obtained when three other species of insects were assayed. *C*. *gleosporioides* has also been shown to have pathogenic activity towards a scale insect. The data suggest that *C*. *acutatum* var. *fioriniae* from *F*. *externa* epizootics in the US, and the previously reported *C*. *gloeosporioides* f. sp. *ortheziidae* causing *Orthezia praelonga* epizootics in Brazil, may constitute distinct biotypes of *Colletotrichum* that have attained the ability to infect insects in addition to the commonly reported plant hosts.

## Introduction

The eastern hemlock, *Tsuga canadensis* (L.) Carrière (Pinales: Pinaceae), a common species in forests of the Northeastern United States is in decline (Orwig et al. 2002). The invasive elongate hemlock scale (EHS), *Fiorinia externa* Ferris (Hemiptera: Diaspididae), has been identified as one of the causal agents of this decline (Lambdin et al. 2005). Attempts to control this pest have not been successful. The unique shield-like cover of the scale provides protection from contact insecticides, natural enemies and adverse climatic conditions. Because of its high reproductive rate, even when mortality exceeding 90% occurs, populations quickly rebound (Baranyovits 1953; Johnson and Lyon 1988).

In 2002 a fungal epizootic, whose geographic point of origin was unknown, was reported within the population of *F*. *externa* in the Mianus River Gorge Preserve in Bedford, NY (McClure 2002). Sclerotia were found concealing the bodies of adult mummified scales. Evidence of this infection was found among scales in 36 different sites in New York, Pennsylvania, Connecticut and New Jersey. A complex of entomopathogenic, phytopathogenic and saprophytic fungi was morphologically and molecularly identified as being associated with the diseased insects (Marcelino et al. 2009a). One species, *Colletotrichum acutatum* var. *fioriniae* var. nov. inedit. (Marcelino et al. 2008), was dominant in this complex and consistently recovered in *F*. *externa* populations in most of the epizootic localities.

Members of the genus *Colletotrichum* are known as cosmopolitan plant pathogens (Sreenivasaprasad and Talhinhas 2005), many of which cause anthracnose in several commercially important crops (Byrne et al. 1997; Lardner et al. 1999; Khan and Hsiang 2003; Horowitz et al. 2004; Xiao et al. 2004). Literature on the phytopathogenic genus *Colletotrichum* (Bailey and Jeger 1992; Prusky et al. 2000) includes a single report of the species *C*. *gloeosponoides* causing significant epizootics in the scale *Orthezia praelonga* Douglas 1891 (Hemiptera: Ortheziidae), a major pest of citrus in Brazil. This fungus, *C*. *gloeosponoides* f. sp. *ortheziidae* is under commercial development for management of *O*. *praelonga* (Cesnik and Ferraz 2000). The epizootic caused by *C*. *acutatum* var. *fioriniae* is the second report of a member of this genus infecting a scale insect. To understand the role of this fungus in the *F*. *externa* epizootic, the virulence of *C*. *acutatum* var. *fioriniae* to four insect species from three orders (Hemiptera, Lepidoptera and Thysanoptera) was evaluated.

## Materials and Methods

### Isolates

The virulence of five *C*. *acutatum* var. *fioriniae* isolates from different areas of the *F*. *externa* epizootic in the Northeast U.S. was tested (Table 1). In addition, the following other isolates were also assayed: *C*. *gloeosporioides* f. sp. *ortheziidae* from the Brazilian epizootic in *O*. *praelonga* (ARSEF4360) (obtained from the Agricultural Research Service Entomopathogenic Fungal Collection, Cornell University, Ithaca, NY), two phytopathogenic *C*. *acutatum*, one isolated from blueberry (ERL1379) and one from tomato (ERL 1380), *Lecanicillium lecanii* (Zimmerman) Gams & Zare (EHS 132), an entomopathogenic fungal strain isolated from *F*. *externa* and one *Metarhizium anisopliae* (Metschn) Sorokin (CA-1), recovered from litter in a California avocado orchard (used only in the *Frankliniella occidentalis* bioassays because of its virulence to this insect). The initial isolation of the fungi was obtained by growth on potato dextrose agar medium (39 g/1) supplemented with penicillin (5 ml/1) and streptomycin (12.5 ml/1). Fungal isolates have been deposited at the University of Vermont Entomology Research Laboratory (UVM ERL) Worldwide Collection of Entomopathogenic Fungi, Burlington, VT. Isolates have been stored as mature mycelium (2 weeks old) in potato dextrose agar cubes (1 cm^2^) in cryogenic vials (8 replicates) containing 10% glycerol and held at -80° C.

Fungi used in the bioassays were grown in potato dextrose agar (39 g/l) for 10–12 days before being harvested with sterile Pasteur pipettes to obtain inoculum suspensions, in sterile distilled water. Calibration of conidial spore suspensions to 10^6^ and 10^7^ conidia/ml^-1^ , concentrations commonly used in insect inoculation bioassays (Abe and Ikegami 2005; Fransen 1987; Klingen et al. 2002), was done using an Improved Neubauer haemacytometer (Propper^®^) according to the protocol of Goettel and Inglis (1997).

### Insects

The virulence of the above fungal isolates was tested for the two increasing doses of inoculum against several insect species representing three orders, Hemiptera, Thysanoptera and Lepidoptera, to understand their comparative infectiveness and dose-mortality response.

The silverleaf whitefly, *Bemisia argentifolii* Bellows and Perring (Hemiptera: Aleroydidae), was reared on poinsettia, *Euphorbia pulcherrima* Wild.ex Klotsch (Malpighiales: Euphorbiaceae) at the UVM ERL according to the protocol of Negasi et al. (1998). For these bioassays, terminal leaves of 2–3 week old bean plants, *Phaseolus vulgaris* L. (Fabales: Fabaceae) var. Royal Burgundy, were excised and the petiole placed in an Oasis^®^ rooting cube (Smithers-Oasis, www.smithersoasis.com) held in place by absorbent cotton wool. Cubes were placed in tap water in 9 mm diameter Petri dishes until roots formed (∼4 days), then 18 mating pairs of whiteflies were removed from poinsettia, anesthetized for 2 seconds with carbon dioxide and placed on each bean leaf. Infested leaves were held in vented plastic boxes (8.7 cm wide × 9.5 cm long) at 16:8 LD, 75% RH and 24°C. Adults were removed after 24 h to ensure age homogeneity of the progeny. On each leaf 40–180 1^st^ instars were produced.

**Table 1.**
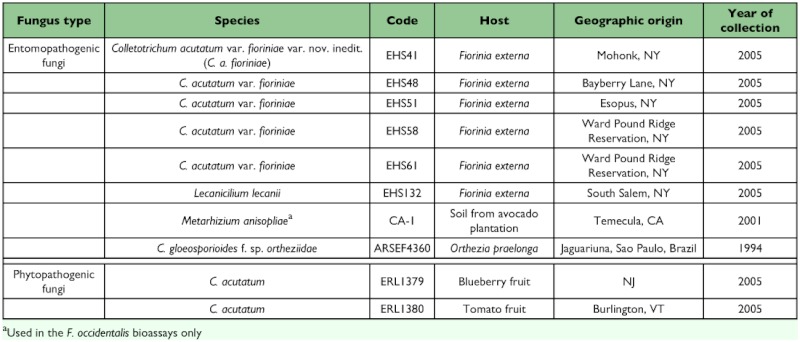
Fungal isolates tested in the insect bioassays

Elongate hemlock scales, *Fiorinia externa* Ferris (Hemiptera: Diaspididae), were field-collected from understory eastern hemlock, *T*. *canadensis* trees at the Mount Tom Forest Preserve, Holyoke, MA, which is located outside the known area of the *F*. *externa* epizootic. One day prior to the bioassay, 30 branches (50 cm long) with new growth and naturally infested with a healthy population of *F*. *externa* crawlers (i.e. 1^st^ instar mobile nymph stage emerged from the 3^rd^ instar adult female exuvia) and settlers (i.e. 2^nd^ instar immobile nymph stage after inserting stylets in the epidermal cells of hemlock leaves, losing their legs and remaining anchored for life) were randomly sampled. Branches were kept cool during transport and held at 4° C prior to treatment. Eighty 10 cm long terminal twigs with new growth were clipped from the branches for the assay. On each infested twig 10–200 settlers and 1–46 crawlers were counted. Freshly pruned branches were gathered for each assay repetition.

Western flower thrips, *Frankliniella occidentalis* Pergande (Thysanoptera: Thripidae), were reared at the UVM ERL on bean leaves (var. Royal Burgundy) according to the protocol of Doane et al. (1998). Two-day-old 2^nd^ instars were used for testing due to the natural high mortality of 1^st^ instars. Each replicate of the assay had 10 thrips/leaf.

Eggs of the beet armyworm, *Spodoptera exigua* Hübner (Lepidoptera: Noctuidae), were purchased from Benzon Research, Inc. (www.benzonresearch.com). Upon delivery, eggs were allowed to hatch in a glass container (12 cm diam. × 20 cm high) containing cabbage leaves. The containers were wiped with an antistatic tissue before introduction of eggs. The glass containers were held for 3–4 days at 22° C and 16:8 LD. After eclosion 20 1^st^ instars were randomly selected for each replicate.

### Bioassays

For all bioassays, fungal concentrations of 10^6^ and 10^7^ conidia/ml^-1^ were suspended in sterile distilled water with 0.02% Silwet (Momentive, www.gesilicones.com) as a surfactant. A 0.02% solution of Silwet was used for the controls, with the exception of *F*. *externa* trials where blank controls were used. Each insect bioassay was repeated three times with four replicates for each treatment.

For the *B*. *argentifolii* assays, each treatment consisted of four leaves, each with 40–180 2 day old 1^st^ instars. A Potter Precision Laboratory Spray Tower (Burkard Manufacturing Co. Ltd., www.burkard.com.uk) operating at 0.84 kg/cmwith a 0.25 mm diameter nozzle was used to spray 2.5 ml of the fungal suspension. Mortality was assessed after 30 days. Individual whole insects were first inspected for morphological changes in the cuticle or body, i.e. changes in color or body turgor. In cases where mortality could not be confirmed in this manner, insects were squashed on a glass slide and checked for the presence of hyphae and fungal spores in the hemolymph, as well as, hyphae penetrating through the cuticle.

For the *F*. *externa* assays, isolates were tested against settiers and crawlers using modified protocols of Rose (1990) and Butt & Goettel (2000). For each treatment (i.e. 10^6^ and 10^7^ conidia/ml^-1^), four twigs, each containing ≥ 10 crawlers or settlers, were held vertically in a metal test tube rack and individually sprayed at a distance of 38 cm with 250 µ″l of a microdroplet mist of a conidial suspension using a hand-held plastic spray bottle. Homogeneous distribution, density and size of sprayed droplets were visually assessed. Twigs were allowed to air dry for 2 min, and were then placed individually in sterile graduated 50 ml conical plastic tubes tubes (www.fishersci.com) containing 16 g of sterilized sand (Quikrete, www.quikrete.com) and 7 ml sterile distilled water. Each tube was covered loosely with a cap to allow ventilation. Tubes were placed in plastic bags to prevent desiccation and held at 22° C with 16:8 LD. Mortality of crawlers and settlers was determined 21 days after treatment as described above for the whitefly assay.

For the *F*. *occidentalis* assays, bean leaf discs (3.3 cm diameter) were placed on moist filter paper in 3.5 cm diameter Petri dishes, to which 10–2^nd^ instars were added. Each Petri dish assembly was sprayed with 2 ml of the fungal suspension using a Potter Spray Tower, as described previously. After being air dried for 2 min, Petri dishes were covered and sealed with Parafilm, and held in the dark at 22 ± 1°C. Mortality was assessed 7 days after treatment. Insects that exhibited obvious signs of fungal infection, i.e. displayed an abnormal body color or lacked turgor, and those that did not respond when gently probed with a small insect pin, were considered dead.

*S*. *exigua* were assayed in well plates with 5 × 4 cells (13 mm diameter/cell) (Model #BIO-BA-128, Color-Dec, www.color-dec.it). A 10 mm diameter disc of moist filter paper was placed in the bottom of each well, followed by 10 mm diameter cabbage leaf disc and one 1^st^ instar beet armyworm. Each 20-cell unit was sprayed with 2 ml of the test suspension with a Potter Spray Tower. Cell units were air dried for 2 min, covered with clear plastic wrap and held in an incubator in the dark at 22 ± 1° C. Mortality was assessed after 7 days as described for the *F*. *occidentalis* assays.

To test Koch's postulates (i.e. re-isolation of the test fungi from a diseased host after treatment of a healthy individual), a random subsample of 10 insects from each bioassay was taken. Each species of insect was surface sterilized in 0.01% NaOCl, rinsed in sterile distilled water and placed in a Petri dish on potato dextrose agar with 5 ml/l penicillin and 12.5 ml/l streptomycin. Petri dishes were held in the dark at 22 ± 1° C for 7 days, and then cadavers were examined for the presence of *C*. *acutatum* var. *fioriniae* or the other fungi tested.

### Statistical analyses

Protocols differed among insect species assayed due to differences in sample sizes of *F*. *externa* and *B*. *arsentifolii* that varied according to female fecundity. Therefore, a statistical treatment adjusted for an unbalanced design was used. Variances were not homogeneous (using Levene's test), hence, a Welch's one way ANOVA (unpooled variances) was carried out. Transformation of the data was not required since they were normally distributed as observed by plotting the residuals from the ANOVA. An adjusted pairwise comparison between fungal isolates within a test insect species was made using a post-hoc Tukey-Kramer test. The effect of suspension concentration (10^6^ or 10^7^ conidia/ml^-1^) was determined with an adjusted least square means (LS means). *P* < 0.05 was considered statistically significant. All statistical analyses were performed using SAS^®^ (SAS Institute 1990) and plotted using SPSS^®^ (SPSS Inc. 2005).

## Results

**Figure 1.**
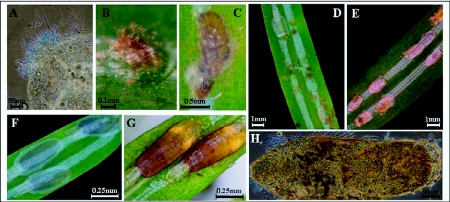
Fungal structures from bioassays of *C*. *acutotum* var. *fioriniae* isolates and symptomatic and non-symptomatic *F*. *externa*. A) acervuli arising from head of an infected whitefly; B) crawler with septicemia from EHS51; C) septicemia in *F*. *externa* adult from EHS41; D) generalized septicemia in crawlers from EHS51 treatment; E) arrested development in diseased *F*. *externa*; F) normal development of the scale cover in *F*. *externa* from controls; G) normal development of the scale cover in nature; H) mycelium arising from adult *F*. *externa*. Bars: A = 50µm; B = 0. 1 mm; C = 0.5 mm; D, E = 1 mm; F, G = 0.25 mm; H = 50 µm.

Definite signs of infection were observed among insects treated with *C*. *acutatum* var. *fioriniae* var. nov. inedit., demonstrating its entomopathogenic capacity (Figure 1A-E). In the control treatments and those sprayed with *C*. *gloeosponoides* f. sp. *ortheziidae F*. *externa* underwent normal development and reached maturity (Figure 1F-G). However, normal development was halted among *F*. *externa* treated with *C*. *acutatum* var. *fioriniae* isolates and phytopathogenic *C*. *acutatum* isolates. Infected *F*. *externa* settlers did not attain maturity (Figure 1E) and both settlers and crawlers showed symptoms of mycosis (Figure 1D-E). Koch's postulate was successfully achieved for the *C*. *acutatum* var. *fioriniae* isolates in *F*. *externa*, silverleaf whitefly and western flower thrips. All 10 cadavers of *F*. *externa*, *S*. *exigua* and *B*. *argentifolii*, and two individuals of *F*. *occidentalis* showed evidence of infection with *C. acutatum* var. *fiorinaiae* (i.e. pink mycelia growing outward from the body of the insects to the media). Infection by *C*. *acutatum* var. *fioriniae* was confirmed by visual examination of spores using a stereomicroscope.

Insect mortality varied depending on isolate, conidial concentration and insect species tested. Differences in mortality between the two conidial concentrations tested (10^6^ and 10^7^ conidia/ml^-1^) were not statistically significant for any of the isolates tested against *F*. *externa* crawlers and settlers whereas for other species tested, *S*. *exigua*, *B*. *argentifolii* and *F*. *occidentalis*, significant differences were observed between the two concentrations tested (Tables 2 and 3).

**Figure 2.**
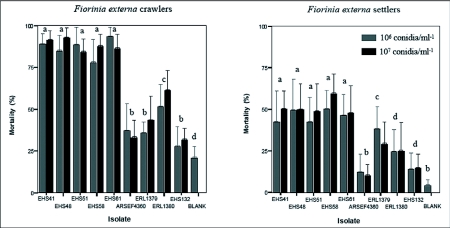
Percent mortality among *F*. *externa* settlers and crawlers in fungal bioassays. (± 95% Cl of the mean). Bars with the same letters are not significantly different (data for both concentrations combined) (*P* < 0.05) using the post-hoc multiple comparison Tukey-Kramer test.

**Figure 3.**
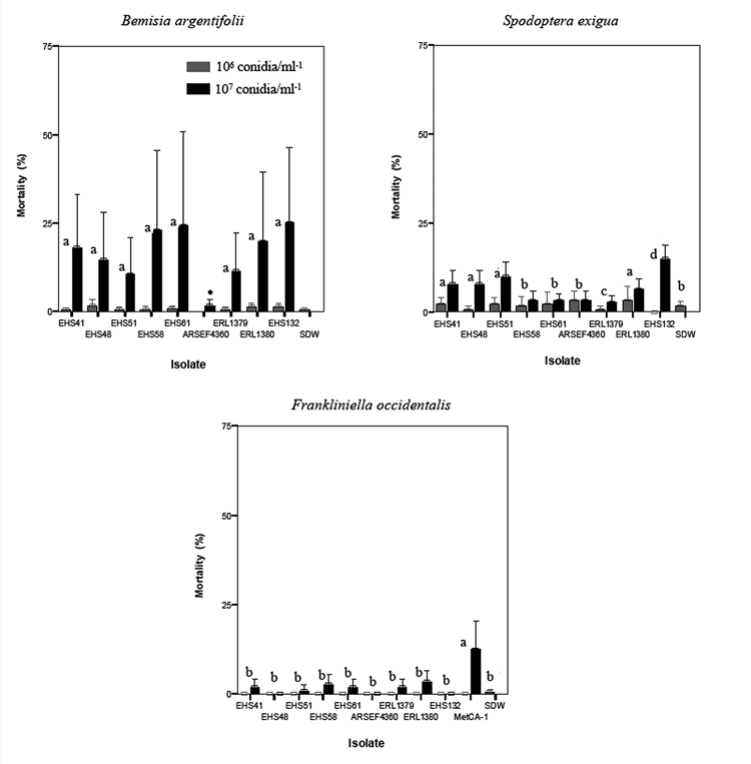
Percent mortality among different test insect species in fungal bioassays. (± 95% Cl of the mean). Bars with the same letters are not significantly different (data for both concentrations combined) (*P* < 0.05) using the post-hoc multiple comparison Tukey-Kramer test. • Statistical significance was not estimated for this isolate because data for only one concentration was collected.

Although a statistical comparison of mortality across species could not be done due to differences in protocols and sample sizes used in the experiments conducted with the four insect species, the mortality caused by *C*. *acutatum* var. *fioriniae* isolates in crawlers and settlers of *F*. *externa* was higher than in the other species tested (Figures 2 and 3). Crawlers of *F*. *externa* were highly susceptible to *C*. *acutatum* var. *fioriniae* isolates (Figure 1D) with a maximum *F*. *externa* crawler mortality rate of 92.64% for isolate EHS48 at 10^6^ conidia/ml^-1^ concentration and 93.44% for isolate EHS61 at 10 conidia/ml-1. When *F*. *externa* settlers were inoculated with isolate EHS58 a maximum mortality rate of 59.27% and 50.28% was observed with 107 conidia/ml^-1^ and 10^6^ conidia/ml^-1^ respectively. In general mortality from *C*. *acutatum* var. *fioriniae* isolates was about 35% greater among crawlers than settlers. For the crawler bioassay, mortality caused by the two other entomopathogens tested, *C*. *gloeosporioides* f. sp. *ortheziidae* ARSEF4360 and *L*. *lecanii* EHS132, was significantly different from all other fungi tested, with the exception of the plant pathogen ERL1379. In contrast, mortality of *F*. *externa* infected with the plant pathogen ERL1380 was significantly greater than the two entomopathogenic fungi ARSEF4360 and EHS132 but always statistically below levels attained with *C*. *acutatum* var. *fioriniae* isolates.

**Table 2.**
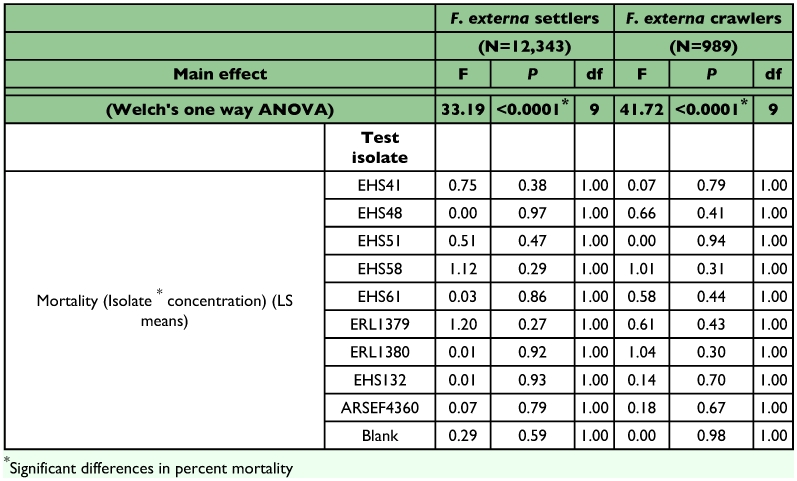
Results of statistical analysis for bioassays with *Fiorinia externa* immatures

Mortality of 22% or less was obtained for the other three insect species tested (*P* < 0.05) with *F*. *occidentalis* and *S*. *exigua* at <10%, and *B*. *argentifolii* between 15 to 22%. However three exceptions were observed: *L*. *lecanii* EHS132 at 107 conidia/ml-1 caused 25% mortality in *B*. *argentifolii* and 14.5% in *S*. *exigua*. *M*. *anisopliae* CA-1 at 107 conidia/ml-1 caused 12.5% mortality in *F*. *occidentalis* (Figure 3). Greater mortality was obtained at the higher conidia concentration. With the exception of *F*. *externa*, whiteflies appeared to be the most susceptible to infection at the higher concentration.

**Table 3.**
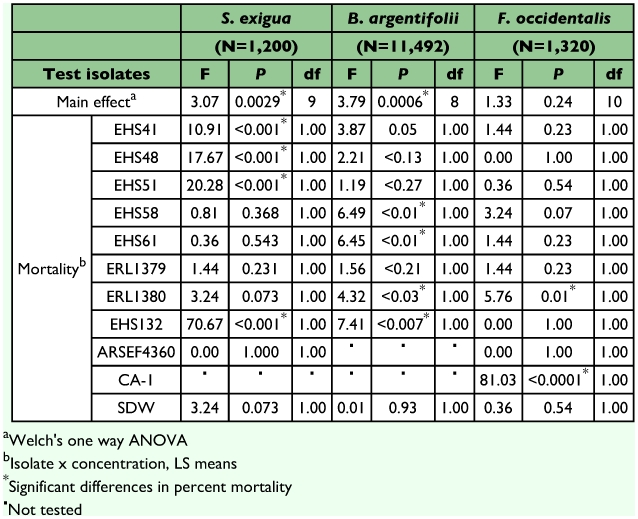
Results of statistical analysis for bioassays with *Spodoptera* *exigua*, *Bemisia argentifolii* and *Frankliniella occidentalis*

## Discussion

This research indicates that the fungus *C*. *acutatum* var. *fioriniae* var.nov. inedit., isolated from infected *F*. *externa* adults recovered from several localities in the North East US hemlock forests where a fungal epizootic occurred was highly pathogenic to this insect host, particularly in the crawler stage.

High mortality rates were caused in both developmental stages of *F*. *externa* infected with inoculations of *C*. *acutatum* var. *fioriniae* var. nov. inedit. Mortality, but at significant lower levels, was also obtained when *F*. *externa* crawlers and settlers were treated with different *C*. *acutatum* isolates known to be phytopathogenic. Mortality in the other three insect species tested with the *C*. *acutatum* var. *fioriniae* and the other fungi was lower: *Bemisia argentifolii* had levels of ≤ 22%, and both *Spodoptera exigua* and *Frankliniella occidentalis* had levels <10%. These results indicate that the latter three species displayed lower susceptibility to both entomopathogenic and phytopathogenic *Colletotrichum* isolates.

This report of virulence of *C*. *acutatum* var. *fioriniae* to *F*. *externa* along with the demonstrated virulence and biocontrol activity of *C*. *gloeosponoides* f. sp. *ortheziidae* to scales of citrus for the last 20 years (Cesnik and Ferraz 2000) supports the generally held hypothesis that members of the genus *Colletotrichum* have a broader host range and inhabit niches other than those currently reported (Guerber and Correll 2001, Peres et al. 2005). The pattern of infection of *F*. *externa* with *C*. *acutatum* var. *fioriniae* has a patchy but widespread geographic distribution. The infection has been detected in the northeastern states of Connecticut, New York, New Jersey and Pennsylvania, suggesting that this infection is established in *F*. *externa* populations in the Northeastern U.S. While the number of insect species tested was limited it appears that the entomopathogenic activity of *C*. *acutatum* var. *fioriniae* was higher against *F*. *externa* than against the other insects tested, suggesting that this variety of *C*. *acutatum* may preferentially infect scale insects. Arthropods from additional orders (Araneae, Hymenoptera, Lepidoptera, Orthoptera) collected from the trunks of hemlocks occurring within the epizootic areas showed no evidence systemic infection with *Colletotrichum* spp. Specimens belonging to the above orders were surface sterilized (following the procedure mentioned herein) and placed on potato dextrose agar did not show evidence of *Colletotnchum* spp. mycelia growth (Marcelino et al. unpublished data).

*C*. *acutatum* var. *fioriniae* has been found growing endophytically in over 28 different species of plants within the epizootic areas. In addition, we have conducted laboratory tests to access the pathogenicity of this fungus to several plants, i.e. strawberries, beans, peppers, tomato, barley and hemlock (Marcelino et al. 2009b). Since this fungus is commonly found in a variety of plants within the Northeast hemlock forest it is possible that this fungus is a native species and that the invasive *F*. *externa* became infected subsequent to its arrival. An alternative hypo-thesis may be that introduced specimens of *F*. *externa* were infected with this variety of fungus at the time of introduction and that both scale and fungus are invasive species.

Mutualist or commensal endophytic associations between plants and members of the genus *Colletotrichum* have been reported (Zulfiqar et al. 1996; Makowski and Mortensen 1998; Tsror and Johnson 2000; Lu et al. 2004). Several similar reports have also been published regarding *C*. *acutatum* (Zulfiqar et al. 1996; Freeman et al. 2001; Horowitz et al. 2002). It has been suggested that the life strategies adapted by *Colletotrichum* spp. (i.e. mutualism, parasitism or commensalism) are controlled in part by the host plant genotype (Redman et al. 2001) and that both chemical and physical factors between the fungus and plant host, direct gene expression of the fungus (Memmott et al. 2002; Dickman 2000, 2004). Similar relationships may have occurred between *F*. *externa* and *C*. *acutatum fioriniae*. Studies with *C*. *magna* endophytic mutants showed that strains with nonpathogenic life strategies had a broader host range than the parental pathogenic *C*. *magna* strains (Redman et al. 2001). *C*. *acutatum* also displays endophytic activity, in most plants we have sampled in the field and tested in the laboratory. This non-pathogenic behavior may have facilitated the shifting of hosts, of this *C*. *acutatum* variety, from plants to insects.

It is apparent from these reports that members of the genus *Colletotrichum* display a great degree of plasticity in host choice, however, to date, most of this work has focused solely on plants. Our data as well as that of Cesnik and Ferraz (2000) provides strong support that members of this genus can also be effective primary pathogens of insects.
